# Nasoalveolar Molding in Cleft Care—Experience in 40 Patients from a Single Centre in Germany

**DOI:** 10.1371/journal.pone.0118103

**Published:** 2015-03-03

**Authors:** Andrea Rau, Lucas M. Ritschl, Thomas Mücke, Klaus-Dietrich Wolff, Denys J. Loeffelbein

**Affiliations:** Department of Oral and Maxillofacial Surgery, Technische Universität München, Munich, Germany; Medical University of South Carolina, UNITED STATES

## Abstract

Nasoalveolar molding (NAM) has gained wide acceptance and evidence in cleft therapy. However, standardized treatment protocols and experiences recorded from European centres are lacking. The results of 40 infants with cleft lip and palate treated with presurgical NAM according to the Grayson technique were analyzed. Standardized parameters of cleft width and nasal symmetry were measured in pre- and posttreatment plaster casts and in digitalized 3-dimensional STL models. Statistical analyses were performed by using Student´s t-test in a per-protocol manner. 27 out of 40 infants completed NAM and were analyzed. In 13 patients NAM was either temporarily interrupted or terminated prematurely due to skin irritations or lack of parental support. These cases were excluded from statistical analysis, resulting in a drop-out rate of 32.5%. Intersegmental alveolar distance (ISAD), intersegmental lip distance (ISLD), nostril height (NH), nostril width (NW) and columella deviation angle (CDA) were significantly changed in unilateral cleft lip and palate (UCLP) (n = 8). In unilateral cleft lip (UCL) (n = 9), only ISLD, NH and CDA were significantly changed. ISAD of the right and left side, ISLD of the right and left side, premaxilla deviation angle, nostril height and columella length were changed significantly in bilateral cleft lip and palate (BCLP) cases (n = 10). NAM is a suitable presurgical treatment modality. A positive effect has been seen in UCLP and BCLP infants, as compared with their birth status.

## Introduction

Various pre- and postsurgical orthodontic and orthofacial techniques have been introduced over the past few decades, in order to overcome the problems associated with wide unilateral cleft lip (UCL), unilateral cleft lip palate (UCLP) or bilateral cleft lip palate (BCLP). Among others, these techniques include oral pinning and traction, pinned co-axial screw, advancement of the cleft maxillary segment, nasal stenting, nasoalveolar molding (NAM) and modifications of NAM [1–4]. NAM was introduced into cleft care and first described by Grayson in the early 1990s [4]. It is based on the high degree of plasticity in the cartilage of neonates due to transient high levels of oestrogen and hyaluronic acid in the early postnatal period [5]. In cleft care, optimal timing of the treatment steps is essential to optimize functional and aesthetic outcome [1,6,7]. Unfortunately, high-level evidence-based studies in the field of NAM are still rare, although a trend towards a significantly positive long-term effect is seen in its application [8,9]. Whereas the technique is relatively well known in the U.S. [10], only a very small number of cleft centres in Europe apply NAM on a regular basis to date.

This study has focused on the analysis of treatment outcomes and of the efficacy of our protocol in terms of narrowing the cleft width and shaping the nasal symmetry before primary surgery. The study has also aimed to compare treatment results of NAM in various types of clefts. Furthermore, we wish to share our experiences of the organizational aspects of integrating NAM into an existing treatment protocol of a cleft care centre in Germany.

## Methods

**Fig 1:**
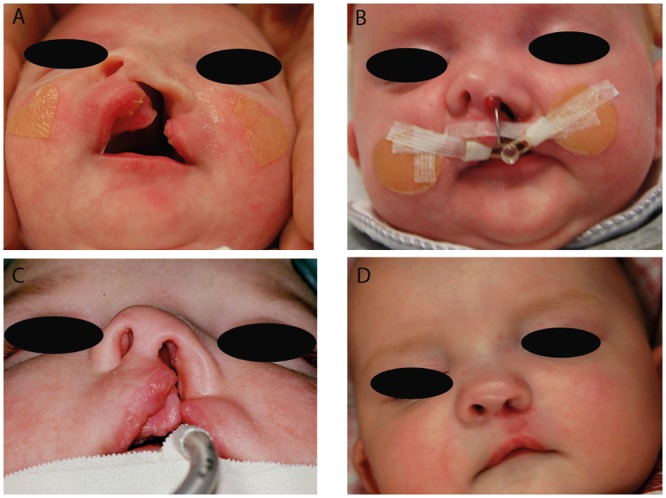
Example for treatment results by using NAM in an infant presenting with a UCLP. (A) preoperative and pre-NAM situation, (B) preoperative situation at a late stage of NAM, (C) preoperative situation with accomplished NAM and (D) postoperative situation three weeks after primary cleft lip repair (published with parental consent).

**Fig 2:**
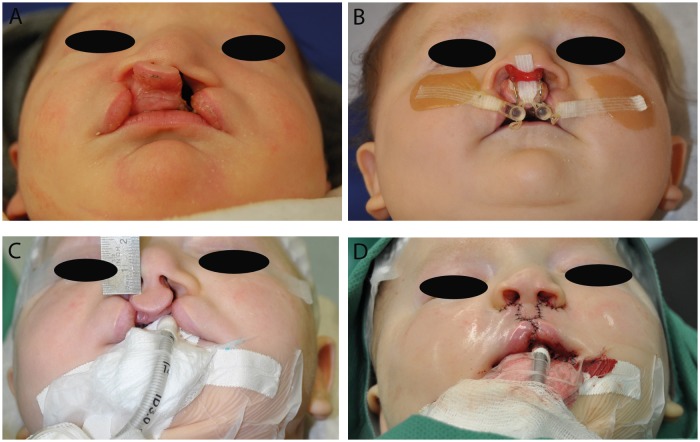
Example for treatment results by using NAM in an infant presenting with BCLP. (A) preoperative and pre-NAM situation, (B) preoperative situation at an early stage of NAM, (C) preoperative situation with accomplished NAM and (D) postoperative situation (published with parental consent).

**Fig 3:**
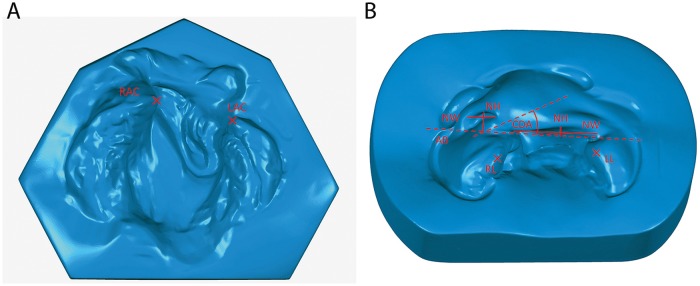
Intraoral (A) and extraoral (B) measuring points in UCLP presented in a 3-dimensional scan of plaster models. (Abbreviations: NW = Nostril width, NH = Nostril height, AB = Alar base, CDA = Columella deviation angle, RL = Right lip, LL = Left lip, RAC = Right alveolar crest, LAC = Left alveolar crest.)

**Fig 4:**
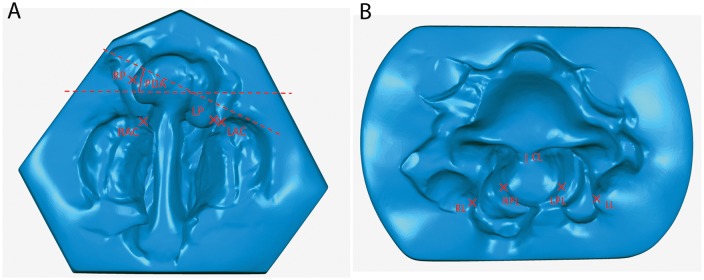
Intraoral (A) and extraoral (B) measuring points in BCLP presented in a 3-dimensional scan of plaster models. (Abbreviations: CL = Columella length, RL = Right lip, LL = Left lip, RPL = Right premaxillary lip, LPL = Left premaxillary lip, RP = Right premaxillary margin, LP = Left premaxillary margin, PDA = Premaxilla deviation angle, RAC = Right alveolar crest, LAC = Left alveolar crest.)

The study has been reviewed by the Ethics committee of the medical faculty, technical university munich (TUM). It has been approved from an ethical and legal point of view. Between March 2010 and November 2012 we offered NAM according to the Grayson technique to the parents of 40 neonates with UCL, UCLP (Fig. 1) or BCLP (Fig. 2) deformities as an optional presurgical orthofacial treatment. In other cases, NAM was not offered because of the following aspects: cleft palate only, age of the child > 6 months of age at initial presentation, consultation from abroad merely for the operation or medical contra-indication for out-patient treatment. After informing the parents concerned and obtaining their written consent, impressions of the cleft-lip-palate-nose complex were taken within two days after birth by using silicone impression materials [11]. NAM started within seven days after birth according to an individual treatment plan depending on the cleft type. For UCLP and BCLP the treatment protocol consisted of two main parts: a period of alveolar molding (duration 6–8 weeks) and a subsequent period of nasal molding (duration 6–8 weeks) after the main correction of the greater alveolar segment was achieved. Nasal molding in BLCP also included columella lengthening. Treatment was continued until primary lip-nose repair. For each patient, a custom-made acrylic plate (Orthocryl^®^, Dentaurum GmbH & Co KG, Ispringen, Germany) covering the palate and alveolar ridge was fabricated. Fixation of this molding plate was achieved by an extraoral taping technique (Figs. 1b and 2b). In addition, lip taping was used for narrowing the lip gap. Once a week the molding plate was modified by the application of acrylic resin (Pattern Resin LS^®^, G.C.) on certain parts and the grinding of other parts to achieve movement and guided growth of the alveolus into the desired direction. Careful examination of the patient was performed regularly to detect pressure marks or other irritation of the skin or mucosa. After 6–8 weeks of alveolar molding, when the alveolar cleft was narrowed to 6 mm or less, a kidney-shaped nasal stent extending from the molding plate was added to lift the alar dome and form the alar wing. This stent was consistently adjusted and activated in order to lift up the lower lateral cartilage and improve nasal symmetry. In BCLP, special attention was given to widening the narrow alveolar arch and to aligning the premaxillary segment into the alveolar arch. Two nasal stents in combination with a specific Y-shaped taping technique were used for simultaneous nasal shaping and columella stretching. In cases of UCL with no affect of the palate, alveolar molding was not necessary and therefore only lip taping was performed during the first six weeks. From the seventh week on nasal molding was performed in the manner described above. Primary lip repair was usually performed at the age of 12–16 weeks, by using a modified Millard’s technique in all cases. Three weeks after the operation, a nostril retainer (Nostril Retainer^®^, Koken CO.LTD., Tokio, Japan) was inserted and fixed via perinasal taping to stabilize the surgical result and to optimize the shaping of the nostrils during further growth. Documentation of the treatment results was performed by taking photographs and impressions at intervals of three weeks from birth to surgery [11]. Standardized parameters of cleft width and nasal symmetry were measured in pre- and posttreatment plaster casts with the help of a sliding calliper and a compass according to other workers [12,13]. For reliability reasons, two examiners performed the measurements independently of one another and mean values were calculated. In addition, the plaster casts were digitalized with a 3D scanner (3Shape A/S^®^, Copenhagen, Denmark). This scanner is equipped with two cameras with a resolution of 1.3 Mega Pixels and a triaxial joint rotation system. Virtual measurements of the digitalized models were performed by using the commercial software package Geomagic Study 12 and Qualify 12^®^ (Geomagic^**©**^, Morrisville, NC, USA) [11] (Figs. 3 and 4). The same observers who performed the manual measurements also performed the digital measurements independently of one another and mean values were calculated. Manual and virtual measuring methods were compared.

The following parameters were measured for pre vs. post NAM situations:

- Intersegmental alveolar distance (ISAD): distance from the right (RAC) to the left alveolar crest (LAC) or to the corresponding premaxillary margin
- Intersegmental lip distance (ISLD): distance from the right (RL) to the left lip (LL) segment or to the corresponding premaxillary lip margin
- Nostril height of the cleft (NHc) and non-cleft side (NHnc): distance from the highest point of the nostril to the alar base (AB) line (connecting line between the right and left alar base)
- Nostril width of the cleft (NWc) and non-cleft side (NWnc): distance from the point farthest right to the point farthest left of the nostril
- Columella deviation angle (CDA): angle between the columella axis and alar base line (only in UCL and UCLP)
  - Premaxilla deviation angle (PDA): angle between the premaxilla axis and a vertical line to the vomer (only in BCLP)
- Columella length (CL): distance from the base of the nose to the highest point of the columella (only in BCLP)

Statistical analyses were performed in a per-protocol manner by using SPSS 20.0 (SPSS, Inc, Chicago, U.S.). Pre- and posttreatment results were compared by using Student’s t-test for dependent samples. Student’s t-test for independent samples was applied for group comparisons of various cleft types. The level of significance was set at p<0.05.

## Results

In the reported period, 40 patients started NAM. Cleft type distribution was as follows: 10 patients with BCLP, 18 patients with UCLP and 12 patients with UCL. 25 patients were male and 15 female. In five cases, the occurrence of mild skin irritations or minor mucosal ulcerations caused a temporary interruption of the therapy. In eight cases NAM was terminated prematurely because of the lack of parental support. Documentation of these cases was not possible, since the patients did not keep their appointments for impressions and photographs. These cases were excluded from the analysis. Twenty-seven patients completed the entire treatment until primary lip surgery (n = 27) without interruptions.

Results for UCLP deformities are shown in Table 1 and Fig. 1 (n = 8). In comparisons of pre- and posttreatment results, statistically significant differences were found for all parameters.

**Table 1:** Changes of pre- and posttreatment cleft parameters in UCLP (n = 8).

Measurement Parameter [mm]	Results mean (SD)	p-Value
ISAD	- 8.13 (5.03)	0.003
ISLD	- 7.5 (4.14)	0.001
NH c	+ 2.86 (0.9)	0.01
NH nc	+ 1.00 (1.16)	0.062
NW c	- 2.57 (1.27)	0.002
NW nc	- 1.00 (1.53)	0.134
CDA	+ 30.29 (14.65)	0.002

**Abbreviations:** ISAD = Intersegmental alveolar distance, ISLD = Intersegmental lip distance, NH = Nostril height, NW = Nostril width, CDA = Columella deviation angle, c = Cleft side, nc = Non-cleft side

Results for UCL deformities are shown in Table 2 (n = 9). In comparisons of pre- and posttreatment results, statistically significant differences were found for all parameters, except for nostril width (p = 0.219).

**Table 2:** Changes of pre- and posttreatment cleft parameters in UCL (n = 9).

Measurement Parameter [mm]	Results mean (SD)	p-Value
ISLD	- 3.87 (3.36)	0.014
NH c	+ 2.11 (1.45)	0.002
NH nc	+ 0.56 (0.53)	0.013
NW c	- 0.89 (1.36)	0.219
NW nc	0	1
CDA	+ 21.11 (12.87)	0.001

**Abbreviations:** ISLD = Intersegmental lip distance, NH = Nostril height, NW = Nostril width, CDA = Columella deviation angle, c = Cleft side, nc = Non-cleft side

Results for BCLP deformities are shown in Table 3 and Fig. 2 (n = 10). In comparisons of pre- and posttreatment results, statistically significant differences were found for all parameters.

**Table 3:** Changes of pre- and posttreatment cleft parameters in BCLP (n = 10).

Measurement Parameter [mm]	Results mean (SD)	p-Value
ISAD r	- 3.6 (2.27)	0.001
ISAD l	- 4.6 (3.1)	0.001
ISLD r	- 1.7 (1.7)	0.012
ISLD l	- 2.00 (2.21)	0.019
NH r	+ 2.7 (1.34)	< 0.001
NH l	+ 1.8 (0.79)	< 0.001
NW r	+ 0.3 (1.57)	0.56
NW l	- 0.5 (1.51)	0.322
PDA	- 9.5 (6.36)	0.001
CL	+ 2.7 (1.06)	0.01

**Abbreviations:** ISAD = Intersegmental alveolar distance, ISLD = Intersegmental lip distance, NH = Nostril height, NW = Nostril width, CDA = Columella deviation angle, PDA = Premaxilla deviation angle, CL = Columella length, l = left side, r = right side

Comparing pre- and posttreatment results of UCLP and UCL (Table 4), for all parameters the extent of change was found to be higher in the UCLP group, but significance was only found for the reduction of nostril width.

**Table 4:** Comparison of posttreatment changes of cleft parameters in UCLP (n = 8) and UCL (n = 9).

Measurement Parameter [mm]	Results mean (SD)UCLP	Results mean (SD)UCL	p-Value
ISLD	- 7.5 (4.14)	- 3.87 (3.36)	0.076
NH c	+ 2.86 (0.9)	+ 2.11 (1.45)	0.229
NH nc	+ 1.00 (1.16)	+ 0.56 (0.53)	0.373
NW c	- 2.57 (1.27)	- 0.89 (1.36)	0.024
NW nc	- 1.00 (1.53)	0	0.134
CDA	+ 30.29 (14.65)	+ 21.11 (12.87)	0.215

**Abbreviations:** ISLD = Intersegmental lip distance, NH = Nostril height, NW = Nostril width, CDA = Columella deviation angle, c = Cleft side, nc = Non-cleft side

No significant differences in measurements were observed, whether the reference was the plaster cast or the digitalized model, by two simultaneous observers.

## Discussion

The evaluation of NAM treatment results requires a detailed 3D registration and imaging of growth changes of the cleft-lip-palate-nose complex. The method has to be quick, precise and harmless to the child. We therefore favor the use of A-silicone based impression materials, since they have a short processing time and an optimal medium viscosity, which guarantees an exact impression. Furthermore, the transparency of the material allows simultaneous optical control [11]. This is especially important when taking impressions of soft tissue areas without bony support. Fabricated plaster casts allow equally well manual and virtual analysis. Surface stereophotogrammetry technology is an alternative method to capture the 3D surface geometry and texture of a face [14,15]. In this technique a random light pattern is projected on to the subject and captures an entire facial area in 0.002 seconds. The short recording time makes it also appropiate for the usage in babies. 3D digital stereophotogrammetry has the advantages of being noninvasive and having a submillimeter accuracy, but until now it cannot be used for intraoral imaging.

Results of the presented study demonstrate that a significant reduction of cleft lip width and alveolar gap was achieved in UCLP and BCLP. Furthermore, the columella axis was significantly uplifted in UCLP, accompanied by a significant reduction of nostril width and a significant increase of nostril height on the cleft side. In contrast to the results of others, we also achieved a significant reduction of nostril width and increase of nostril height in unilateral clefts [16,17]. In combination, these changes contribute to a better nasal shape and symmetry in UCL and in UCLP. The good results of nasal symmetrization in our study might be attributable to the delayed start of nasal stenting, according to Grayson and Suri et al [6,12,18]. Furthermore, we try to integrate a retention period of at least one week after slight overcorrection of the nostril height before primary lip-nose repair. No primary rhinoplasty was performed; only soft tissue medialization of the alar base was performed according to Millard’s technique. In the comparison between UCL and UCLP, the UCLP group showed a greater extent of change of all parameters, although significance was only found for the reduction of nostril width. Different starting conditions, namely complete cleft configuration with complete non-integrity of the nasal floor and therefore wider clefts might be the reason for better reduction of the nostril width in UCLP than in UCL. In the presented study, we recognized the significant elongation of the columella length in infants with BCLP as reported by others (p < = 0.01) [19–21]. Additionally, our results showed a significant elongation and significant rotation of the premaxilla segment in BCLP. According to Spengler et al. the combination of columella lengthening and repositioning with nostril lengthening improves nasal symmetry significantly [21]. Our results imply that NAM helps to improve nasal symmetry and premaxillary alignment in BCLP. The optimal timing for the commencement of NAM remains under debate amongst specialists; this procedure is usually performed preoperatively, shortly after birth until primary lip repair [6,8,22,23]. According to the Grayson technique, the most suitable moment occurs once the distance of the alveolar cleft is narrowed by alveolar molding to 5 mm or less. Commencement at this point and not earlier should avoid undesired lengthening of the alar rim as the initially highly stretched alar rim is more relaxed. In contrast, Figueroa et al. start alveolar and nasal molding simultaneously shortly after birth [24]. When comparing the two techniques, Liao et al. describe that the nostril width is reduced significantly only in the Grayson group [12]. The authors presume that an undesirable increase in the circumference of the lateral alar wall might be a risk factor of an early nasal molding in larger alveolar clefts and might result in a “mega nostril”. Further arguments for preferring a delayed start of nasal molding are mucosal lining trauma, tissue breakdown and notching in the medial angle of the ala [6]. According to our own experience, a period of six to eight weeks is sufficient to lift up the nostril and mold nasal symmetry in most cases. We have not seen the phenomena of mega nostrils after the molding procedure. In order to maintain treatment results stable, a retention period of one to three weeks with the NAM appliance in situ is essential up until the day of surgery. We therefore recommend the insertion of the nasal stent(s) at approximately six to eight weeks after birth when the alveolar gap(s) is/are reduced to 6 mm or less in UCLP and BLCP. In UCL, we start nasal molding with a delay of six weeks to avoid unnecessarily long treatment times. In thirteen out of forty patients NAM was either temporarily interrupted for more than three days or terminated prematurely. According to our study criteria, these cases were excluded from statistical analysis, resulting in a drop-out rate of 32.5%. Our strict exclusion criteria may have lead to this relative high number of drop-outs. Nevertheless NAM was continued after interruption in some of these cases—therefore the term “drop-out” refers to the statistical and not to the therapeutical point of view. Reasons for a temporary interruption of NAM or the premature termination of the treatment were either lack of parental support or child-related reasons such as skin irritations or restlessness. Since the patients were seen by our team only once a week, the parents were responsible for the daily taping procedures. This demonstrates that parental support and compliance is essential and crucial for the success of NAM [4,10]. The incidence of mucosal or skin irritations [4,25] in our study was not that high as that reported by Liao et al. but nevertheless led to the termination of treatment in five cases (12.5%). Lioa et al. have reported the incidence of mucosal ulcerations as being 23% in the Grayson group compared with only 3% in the Figueroa group [12].

## Conclusion

As we integrated NAM into our cleft treatment concept no earlier than 2010, we cannot present long-term results at this point. Initial follow-up investigations are currently being performed. NAM has proved to be an efficient method for reducing cleft width and improving nasal shape and symmetry in uni- and bilateral clefts. Various cleft types and manifestations react differently to the therapy. Digitalization of plaster models for treatment observation and scientific evaluation seem to be an accurate way of documentation.

Despite the differing opinions about the long-term success of NAM, the immediate success of the therapy facilitates cleft surgery immensely. Although lip closure can always be achieved surgically regardless of the cleft width, preoperative narrowing of the lip and alveolar segments, nasal shaping and columella lengthening help to reduce tissue tension and therefore improve surgical outcome by minimizing wound healing disturbances and scarring. For treatment success, a high compliance and active participation of the patient’s parents is however indispensable.
